# Prolonged tamoxifen‐enriched diet is associated with cardiomyopathy and nutritional frailty in mice

**DOI:** 10.1113/EP091668

**Published:** 2024-01-30

**Authors:** Janith Halpage, Patricia DaSilva Pantoja, Salvatore Mancarella

**Affiliations:** ^1^ Department of Physiology University of Tennessee Health Sciences Center Memphis Tennessee USA

**Keywords:** Cre‐lox, recombination, special diet, tamoxifen

## Abstract

Tamoxifen (TAM) is required for gene recombination in the inducible Cre/lox system. The TAM‐enriched diet is considered safe, with negligible impact on animal wellbeing. However, studies reporting the long‐term effects of the TAM diet and its potential impact on experimental outcomes are scarce. We conducted a longitudinal study on mice exposed to a 4‐week dietary TAM citrate supplementation. Several parameters were recorded, such as body weight, body composition, mortality, and cardiac function. The *collagen1a2* (*Col1a2*) transgenic mouse was used to assess TAM‐induced recombination in vivo in cardiac fibroblasts followed by myocardial infarction (MI). The impact of TAM on the MI outcome was also evaluated. The recombination efficiency and cytotoxic effect of the TAM active metabolite, 4‐hydroxy‐tamoxifen (4‐OHT), were assessed in vitro. Mice exposed to a TAM diet showed body weight loss and a 10% increase in mortality (*P* = 0.045). The TAM diet decreased cardiac function and induced cardiac remodeling, indicated by decreased fractional shortening from 32.23% to 19.23% (*P* = 0.001) and left ventricular (LV) wall thinning. All measured parameters were reversed to normal when mice were returned to a normal diet. Infarcted Col1a2‐CreER mice on the TAM regimen showed gene recombination in fibroblasts, but it was associated with a substantial increase in mortality post‐surgery (2.5‐fold) compared to the controls. In vitro, 4‐OHT induced gene editing in fibroblasts; however, cell growth arrest and cytotoxicity were observed at high concentrations. In conclusion, prolonged exposure to the TAM diet can be detrimental and necessitates careful model selection and interpretation of the results.

## Abstract (highlights)

- **What is the central question of this study?**
  Tamoxifen is used to induce Cre‐mediated gene editing, but high doses used in intraperitoneal injection can be toxic and tamoxifen‐enriched food has emerged as an alternative: what is the impact of long‐term exposure to the tamoxifen diet in mice?
- **What is the main finding and its importance?**
  Prolonged exposure to the tamoxifen diet induced severe weight loss, weakness, and impaired cardiac function. Mice exposed to long‐term tamoxifen‐enriched diet are more likely to succumb to further interventions. Investigators planning to use inducible mouse models should be aware of the potential problems that can be avoided with careful experimental design.

## 1 INTRODUCTION

Conventional knockout mouse models allow researchers to study the function of a specific gene in physiological and pathophysiological conditions. However, early deletion of the targeted gene often leads to embryonic lethality, precluding further analysis in mature animals. Furthermore, in these models, the target gene is inactivated in every cell type of the mouse body, making it impossible to investigate the biological significance of a target gene in a particular tissue. The *Cre–loxP* system consists of two mouse lines: one contains a modified genome in which two *loxP* elements are inserted in two introns, flanking the targeted gene's critical exon(s), and the other uses a tissue‐/cell‐type‐specific promoter to express Cre (Bouabe & Okkenhaug, 2013; Littlewood et al., 1995; Nagy, 2000; Ray et al., 2000). The *loxP*‐modified (floxed) gene remains fully functional, except in cells expressing Cre under the control of a tissue‐specific promoter. In a tamoxifen (TAM)‐inducible Cre recombinase mouse model, the Cre recombinase is fused to one or two (CreERT, MerCreMer) mutated oestrogen‐binding domains (Glaser et al., 2005; Indra et al., 1999). Therefore, only in the presence of the TAM active metabolite, 4‐hydroxy‐tamoxifen (4‐OHT), can Cre recombinase induce the inactivation of the lox‐targeted gene (Indra et al., 1999; Metzger et al., 1995). Mice treated with TAM quickly metabolize it to 4‐OHT in the liver, and this is released into the circulation to reach the target cells (Mandlekar et al., 2000). Therefore, combining a cell‐specific promoter and the fusion of a mutant oestrogen receptor to Cre recombinase allows precise spatiotemporal control of gene expression, circumventing issues of embryonic lethality when ablation of the gene of interest affects fetal development.

Many studies have shown that a moderate‐to‐high concentration of TAM is cardiotoxic, causing transient cardiac dysfunction, dysregulated energy metabolism and cardiomyopathy (Koitabashi et al., 2009; Rouhi et al., 2022; Yan et al., 2015; Yoshinobu et al., 2021). In particular, transgenic mice expressing the Cre‐recombinase under the control of the α‐myosin heavy chain gene exhibit DNA damage, focal fibrosis and depressed left ventricular (LV) function after TAM treatment (Lexow et al., 2013). The causes of this detrimental effect remain controversial as they have been attributed to both TAM and the presence of high expression of Cre‐recombinase in the heart (Heinen et al., 2021). However, direct application of TAM reduces cardiac conduction and decreases contractility in a dose‐dependent manner when applied directly to cardiomyocytes (Asp et al., 2013; Xie et al., 2023), further suggesting a direct effect of TAM on the heart. Based on this evidence, there is a great need to understand and refine the use of TAM in laboratory animals.

TAM is highly lipophilic, and it is usually dissolved in ethanol and oil before intraperitoneal (i.p.) injection into the mice. However, chronic peritoneal inflammation has been observed after repeated subcutaneous or i.p. oil injections (Alsina‐Sanchis et al., 2021; Kuroda et al., 2004; Ramot et al., 2009). To mitigate the undesired TAM‐ and oil‐induced side effects (Kiermayer et al., 2007; Yoshinobu et al., 2021), administration of TAM by oral gavage or commercially available TAM‐enriched food has been used as an alternative (Andersson et al., 2010; Kiermayer et al., 2007; Yoshinobu et al., 2021). However, the TAM‐enriched diet showed reduced gene recombination efficiency compared to other TAM delivery methods (Smith et al., 2022). Therefore, mice are typically fed with TAM‐enriched food for an extended period (Fontaine et al., 2013). While occasional food avoidance has been observed (Kiermayer et al., 2007), the impact of long‐term exposure to the TAM diet on animal welfare and cardiac function has not been reported. To date, it is unclear whether TAM provided as a dietary supplement would be a valuable alternative to avoid the detrimental effects caused by the injection of an oil‐based mixture.

Mechanical stress and inflammation stimulate cardiac fibroblast proliferation, promoting differentiation into myofibroblasts (Kanisicak et al., 2016). Prolonged activation of myofibroblasts leads to sustained fibrotic processes within the myocardium, leading to deterioration of heart function. Mouse models expressing fibroblast/myofibroblast‐specific TAM‐inducible Cre‐recombinase have been extensively used to investigate the role of genes of interest in the progression of cardiac fibrosis in models such as chronic hypertension or myocardial infarction (MI). However, the potential influence of TAM on the outcome of these models is usually not included in the reports.

Given the increasing awareness of the detrimental effects of TAM in mice and the lack of data regarding the use of TAM‐supplemented diets, the objectives of this study were to determine the impact of a long‐term TAM diet on a laboratory mouse and whether the TAM‐diet regimen influences the outcome in an experimental model of MI. Gene recombination efficiency and TAM toxicity were evaluated in vivo and in vitro. These studies will inform the development of a better approach for using TAM in Cre‐inducible models and setting up appropriate controls for correct data interpretation.

## 2 METHODS

### 2.1 Ethical approval

The Institutional Animal Care and Use Committee (IACUC) at the University of Tennessee Health Science Center approved all protocols and animal care guidelines (IACUC ID: 22‐0370.0). Procedures were performed in compliance with the Animal Welfare Act and comply with the animal ethics required by *Experimental Physiology*.

### 2.2 Mice

C57BL/6J mice (stock no. 000664), transgenic *Col1a2‐CreER* mice (Stock 029567), and double‐fluorescent reporter mice ROSAmT/mG (ROSA^mT/mG^) mice (stock no. 7676) were purchased from The Jackson Laboratory (Bar Harbor, ME, USA). The Col1a2‐CreER were crossed with ROSA^mT/mG^ to obtain the Col1a2‐CreERT^mT/mG^ mice. This strain allows tracking of Cre‐recombination events by turning Cre‐expressing cells into green fluorescent protein (GFP)‐positive cells. Mice were moved to a biohazard room 3 days before the beginning of the diet and returned to the regular room 3 days after TAM treatment ceased. Rooms were maintained at 22°C with an alternating 12:12 h light–dark cycle. The cage and food were changed once a week. Mice had free access to food and water ad libitum.

### 2.3 Experimental design

C57BL/6J mice of 4–12 weeks old were randomly assigned to one of the two dietary treatments: the control group was fed with a standard rodent chow (Inotiv, no. 7912 Inotiv, Indianapolis, IN, USA), and the treated group was fed with food pellets containing 400 mg/kg tamoxifen citrate (66% tamoxifen, Inotiv, TD.55125) for a total of 4 weeks. Both groups were monitored for body weight, body composition, mortality and cardiac function. After 4 weeks of treatment, 50% of animals from each group were killed for gravimetric analysis. The remaining animals were switched to a regular diet and monitored for 4 weeks. In another set of experiments, Col1a2‐CreERT^mT/mG^ mice 8–12 weeks old were randomized and subjected to a control or a TAM diet. On the fifth day of treatment, the mice received a permanent coronary artery ligation or sham operation. The diet continued for 4 weeks, at the end of which the mice were killed.

### 2.4 Echo magnetic resonance imaging

Body composition was assessed using the EchoMRI‐1100 system (EchoMRI LCC, Houston, TX, USA). Each mouse was placed in an adjustable plastic cylinder to restrict movement. The cylinder (1.25 inches in diameter) had holes on each end to allow for the animal to breathe normally. The animal (cylinder) was inserted into the Echo MRI for 30–75 s to obtain readings that included lean mass, fat mass, total water and free body water (Kuefner et al., 2017).

### 2.5 Echocardiography

Mice were anaesthetized with isoflurane (3–5%) and placed on the warming pad of a recording stage of a Vevo 2100 ultrasound machine. Anaesthesia was maintained with continuous isoflurane at 2%. The anterior chest was previously shaved, ultrasound coupling gel was applied, electrodes were connected to each limb and an ECG was simultaneously recorded while body temperature was monitored. Two‐dimensional M‐mode measurements (at the level of the papillary muscles) were taken using an 18–32‐MHz MS400 transducer. Images were recorded in the short and parasternal long axis. For analysis purposes, ≥3 beats within the same heart rate interval (500 ± 50 bpm) were measured and averaged as previously described (Gammons et al., 2021).

### 2.6 Permanent ligation of the left anterior descending coronary artery

A myocardial injury model was generated on day 5 of introducing the TAM diet. Mice were anaesthetized with isoflurane (3–5%) and attached to a ventilator providing continuous isoflurane at 2%. After surgical preparation, a 1‐cm vertical incision was performed in the midclavicular line for a lateral thoracotomy. Mice were subjected to MI by permanent ligation (8/0 nylon suture) of the left anterior descending coronary artery at about 1 mm below the edge of the left auricle. In the sham group, the coronary artery was not ligated. The chest was closed, and the pneumothorax was reduced. Mice were monitored continuously during recovery until the righting reflex was regained. Post‐operative analgesia (buprenorphine, 0.1 mg/kg) was administered subcutaneously twice daily for 3 days. Non‐invasive echocardiographic evaluation was performed to monitor cardiac function. At the end of the fourth week, post‐MI echocardiography was performed. Mice were later killed, and hearts were collected for further analysis as previously described (Kamatham et al., 2019).

### 2.7 Preparation of single cell suspension from mouse heart

Untreated Col1a2‐CreERT^mT/mG^ mice were used to obtain fibroblast for in vitro assays. Mouse embryonic fibroblasts (MEFs) were obtained from timed pregnant females. Adult mice were anaesthetized with isoflurane (4–5%), followed by cervical dislocation, and embryos were rapidly decapitated. The heart was removed, and the embryonic tissue was washed in cold phosphate‐buffered saline (PBS), minced and digested with DNase and Collagenase type II (Worthington Biochemical Corp., Lakewood, NJ, USA). Cells were collected in fetal bovine serum (FBS), centrifuged and then resuspended in complete Dulbecco's modified Eagle's medium (DMEM) containing 4.5 g/l d‐glucose and 10% FBS, 100 U/ml penicillin, and 100 μg/ml streptomycin at 37°C and 5% CO_2_. Adult cardiac fibroblasts were isolated from 6‐ to 8‐week‐old Col1a2‐CreERT^mT/mG^ mice. The heart was perfused with ice‐cold sterile Hanks' balanced salt solution (HBSS) (Fisher Scientific, Waltham, MA, USA, SH30588.01), cut into small pieces, resuspended in HBSS containing DNase and Collagenase II, and incubated for 45 min at 37°C. Digested tissue was filtered through a 70‐μm cell strainer followed by low‐speed centrifugation (50 *g*, 2 min) to remove cardiomyocytes, cell clumps and the remaining undigested tissue. Next, the supernatants were passed through a 40‐μm cell strainer, centrifuged at 450 *g* for 4 min, and washed with PBS. Cells were plated, and the complete medium was changed after 2 h. For in vitro toxicity assay, cells were grown in 96‐well plates and imaged with an IncuCyte SX5 live‐cell imaging instrument (Essen BioScience Ann Arbor, MI, USA). The relative proliferation/cell death was calculated using IncuCyte analysis software.

### 2.8 Immunofluorescence

Mice were injected with heparin (i.p. injection, 500 IU/mouse) and killed (isoflurane 5% followed by cervical dislocation). The heart was quickly arrested in diastole by nominally Ca^2+^‐free Tyrode solution containing 2,3‐butanedione monoxime (10 mmol/l) and fixed in 10% formalin for 20 min under pressure (diastolic arterial pressure). The heart was excised, weighed, and stored in formalin for 24–48 h. Tissue was processed and embedded in paraffin. Immunostaining against GFP was performed on 5‐μm‐thick paraffin‐embedded tissue sections. To assess the GFP signal with immunostaining, cells were treated with rabbit anti‐GFP antibody (1:500, Cell Signaling Technology, Danvers, MA, USA) as previously described (Parks et al., 2016).

### 2.9 Statistical analysis

All data are represented as means ± standard deviation (SD). Differences between baseline measurements were assessed using Student's paired *t*‐test. One‐way ANOVA and repeated measures ANOVA were used to detect differences across the experimental groups for variable body weight, fat mass, lean mass, free water (%), GFP^+^ cells within the cardiac tissue, and cell confluence in vitro. If significant differences were detected, Tukey's HSD *post hoc* test was performed to explore distinct group differences. The normality of data was assessed using the Shapiro–Wilk test. The Greenhouse–Geisser correction was applied when assumptions of sphericity were violated. Survival endpoint dates were recorded for all mice during the 4‐week TAM exposure and the following 4 weeks when mice returned to the regular diet (recovery time). Survival was analysed with the log‐rank test. All statistical analyses were conducted in GraphPad Prism software (version 9.0.1, GraphPad Software, Boston, MA, USA). All graphs display means ± SD, plotting individual data points; statistical significance was set at *P* < 0.05.

## 3 RESULTS

### 3.1 Body weight variation during TAM‐enriched diet

**FIGURE 1:**
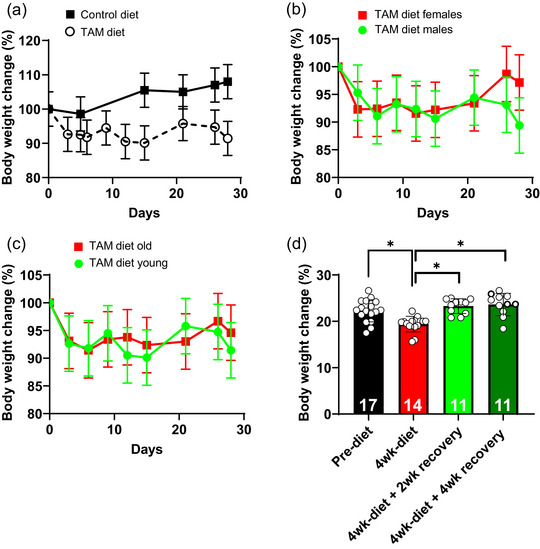
Temporal body weight variation of C57BL/6 mice in response to TAM diet. (a) Percentage body weight changes in mice fed with regular diet (Control group, *n* = 16 males and *n* = 15 females), compared to mice fed with TAM diet (*n* = 18 males, *n* = 17 females) during 4 weeks of treatment. (b) Percentage body weight changes in males (*n* = 18) compared to females (*n* = 17) fed with a TAM‐enriched diet. (c) Percentage of body weight changes in young (1 month ± 2 weeks; *n* = 9) and mature (3 months ± 1 week; *n* = 17) mice. The graph includes both males and females. (d) Body weight changes during TAM treatment and recovery when mice returned to the regular diet. The pre‐diet weight was taken 1 day before TAM treatment started. Mouse weight was recorded 2 and 4 weeks after mice were returned to a standard diet, digits inside the bar represent the mouse number. A repeated measures *t*‐test was used for (a–c), repeated measures two‐way ANOVA followed by Tukey's multiple comparisons test for (d); *P < 0.0001.

Mice were randomized into two groups, including an equal number of males and females of 8–12 weeks of age. The control group (*n* = 31) was fed a regular diet, and the experimental group (*n* = 35) was fed TAM‐enriched pellets for 4 weeks. The body weight for each mouse was measured every 3–5 days. The average trends in body weight and growth rate, stratified by dietary intervention, are shown in Figure 1a. Mice switched to TAM feed lost 7% of body weight within the first 3 days and an additional 3% during the remaining period of the diet (Figure 1a). At the end of the 4 weeks, TAM‐fed mice were 27.6% lighter than their control counterpart. In contrast, mice in the control group gained 10% body weight during the same period. This observation suggests that dietary TAM contributes to body weight loss. A clustered analysis of males (*n* = 18) versus females (*n* = 17) revealed that the TAM chow diet induced similar relative weight loss in both sexes (Figure 1b, *P* = 0.432). In a different cohort, mice segregated by age (young, 1 month ± 2 weeks, *n* = 9 versus mature, 3 months ± 1 week, *n* = 17) exhibited a similar relative body weight loss (Figure 1c, *P* = 0.435). Overall, mice fed with TAM chow for 4 weeks lost significant body weight independent of sex and developmental stage (*P* < 0.0001). Mice that were fed with TAM for 4 weeks and then moved to a regular diet regained their weight within 2 weeks (*P* < 0.0001). At the end of the 4‐week recovery period, the mice's appearance and body weight were similar to the pre‐diet value (Figure 1d).

### 3.2 Body composition analysis during and after TAM‐enriched diet

**FIGURE 2:**
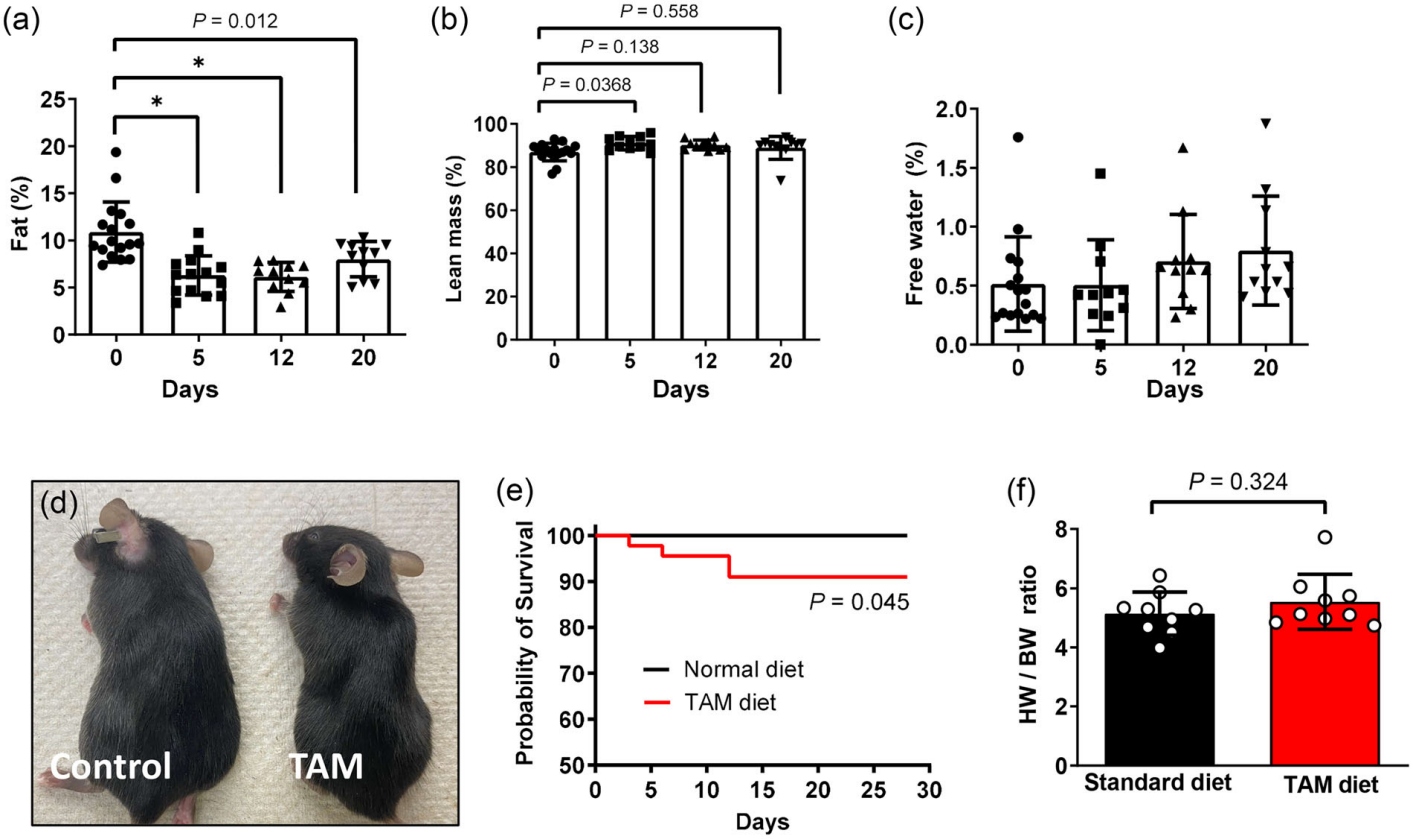
Body composition analysis generated by echoMRI scan. (a–c) Percentage of fat mass (a), percentage of lean mass (b), and percentage of body water (c) from day 0 (*n* = 17), day 5 (*n* = 14), day 12 (*n* = 11) and day 20 (*n* = 11) of TAM diet. (d) Representative image showing isoflurane‐anaesthetized same‐sex littermate mice; the mouse on the left was fed a standard diet (control) and the mouse on the right fed with TAM‐enriched pellets (TAM diet) for 12 days. (e) Kaplan–Meier cumulative survival plots of TAM‐fed mice (*n* = 31) compared with untreated controls (*n* = 35); log‐rank test *P* = 0.045. (f) Heart weight/body weight (HW/BW) coefficient from mice fed with TAM diet for 4 weeks (*n* = 9) compared to control mice (*n* = 9); paired *t*‐test *P* = 0.324.

Body composition analysis performed with EchoMRI™ magnetic resonance imaging revealed that the TAM diet induced a rapid reduction of body fat mass measured at days 5 and 12, with partial recovery at around day 20. Still, it remained significantly lower when compared to the pre‐diet measurement (Figure 2a, *P* = 0.012). The lean mass significantly increased as soon as 5 days after the beginning of the diet and remained stable for the duration of the treatment (Figure 2b, *P* = 0.036). The body content of free water did not change in these animals (Figure 2c, *P* = 0.096). TAM‐treated animals showed the clinical phenotype of body mass loss (Figure 2d). About 10% of animals died within the first 2 weeks since the beginning of the diet, and there were no deaths after this initial interval (Figure 2e, *P* = 0.045). The heart‐to‐body weight ratio (HW/BW) was unchanged after 4 weeks of treatment (Figure 2f).

### 3.3 Effect of TAM oral administration on cardiac function and anatomy

**FIGURE 3:**
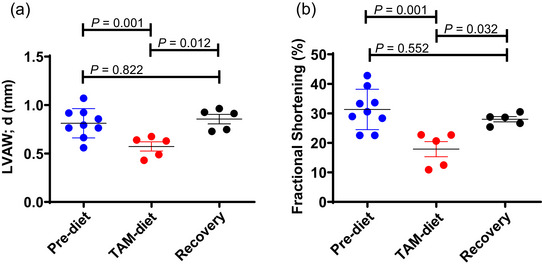
Echocardiographic analysis of mice fed with TAM diet. Echocardiography was performed on the same mice 1 day before the TAM diet (pre‐diet, *n* = 9), 4 weeks after the TAM diet (TAM diet, *n* = 5) and 4 weeks after the mice returned to the normal diet (recovery, *n* = 5). (a) Diastolic LV anterior wall thickness. (b) Fractional shortening (FS%). One‐way ANOVA followed by Tukey's multiple comparisons test was used.

The causes of the detrimental effect on the heart remain controversial as they have been attributed to both TAM and high expression of Cre‐recombinase in the heart (Heinen et al., 2021). We tested whether oral consumption of TAM and sustained body weight loss were associated with changes in cardiac function and heart anatomy. A comprehensive echocardiographic evaluation showed that 4 weeks of TAM treatment resulted in a thinner left ventricular anterior wall (LVAW) compared to the pre‐diet values (Figure 3a, *P* = 0.001). Mice left to recover on a regular diet for 4 weeks showed that the LVAW reversed its pre‐diet values. Cardiac function assessed as fractional shortening (%FS) was significantly depressed by the TAM diet compared to the pre‐diet values (Figure 3b, *P* = 0.001). As with the anatomical data, mice that were returned to a regular diet regained their original cardiac contractility (Figure 3b).

### 3.4 TAM‐diet effect on gene recombination and outcome in an MI model

**FIGURE 4:**
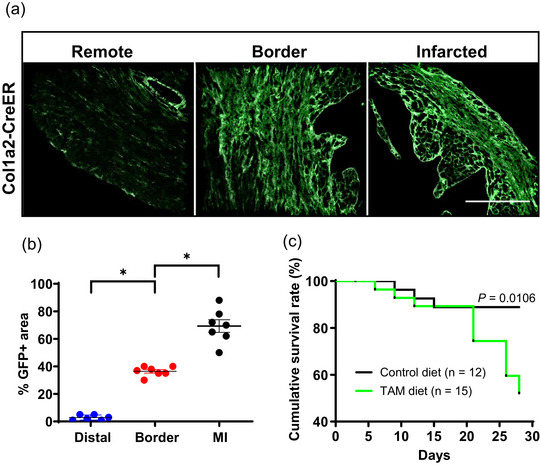
Assessment of Col1a2‐CreER‐driven Cre‐recombinase activity in infarcted hearts. (a) Representative immunofluorescence images of GFP staining on heart sections from Col1a2‐CreER^mT/mG^ after 28 days of MI injury and TAM diet. Heart tissue was stained with rabbit anti‐GFP antibody. Representative images were extracted from the distal, border and MI regions (scale bar: 500 μm). Each image is representative of five individual mouse samples. (b) Quantification analysis of GFP^+^ areas from the remote zone, the infarcted border zone and the infarcted tissue. Sample size: distal *n* = 6, border *n* = 7, and infarcted *n* = 7 slides extracted from five animals. One‐way ANOVA followed by Tukey's multiple comparisons test; *P < 0.0001. (c) In the Kaplan–Meier cumulative survival plot and log‐rank test, mice fed with a control or TAM diet underwent MI surgery.

TAM induces Cre‐mediated gene recombination to silence the targeted gene in vivo. We tested whether a TAM‐enriched diet was sufficient to induce Cre expression and gene recombination in a mouse model of MI. For this purpose, a Col1a2 Cre‐ER mutant mouse carrying the membrane Tomato/membrane GFP (mTmG) fluorescent reporter was used to test TAM‐induced gene recombination in cardiac fibroblasts 28 days post‐injury. The most distal region to the infarcted area, named the ‘remote zone’, showed negligible GFP^+^ signal. The ‘border zone’ surrounding the infarcted area and the infarcted tissue that is rich in fibroblast‐secreting collagen (Lal et al., 2014) showed the presence of GFP^+^ cells, suggesting TAM's effectiveness in inducing gene recombination (Figure 4a,b, *P* = 0.0001). About 50% of the mice receiving both MI and the TAM diet died prematurely during the 4 weeks post‐surgery (Figure 4c, *P* = 0.0106). These results suggest that while a TAM‐supplemented diet successfully induces gene recombination in the infarcted heart, combining the TAM diet with additional stress, such as coronary artery ligation, significantly increased mortality rates.

### 3.5 Analysis of 4‐OHT toxicity in vitro

**FIGURE 5:**
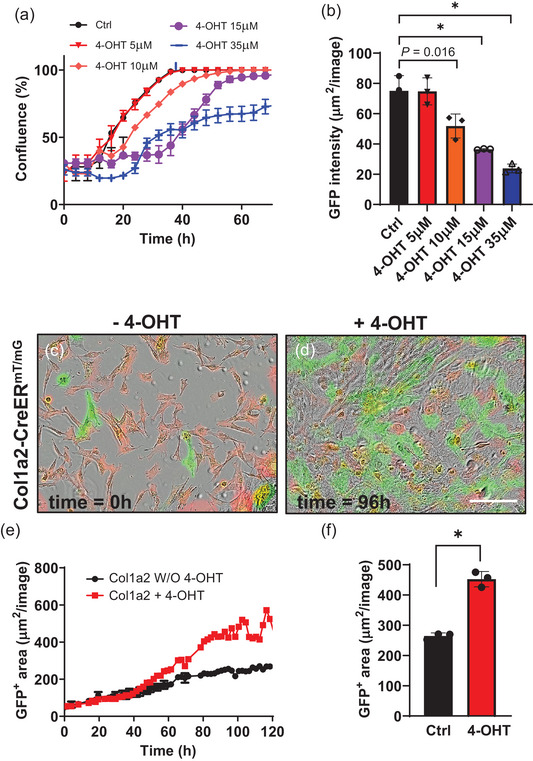
Genetic recombination induced by 4‐OHT treatment in cultured Col1a2‐CreER^mT/mG^ MEFs. (a, b) Cells were treated with incremental doses of 4‐OHT, and longitudinal changes in cell confluence (%) were observed over 4 days. (c, d) Representative live‐cell images of Col1a2‐CreER^mT/mG^ MEFs before and after (96 h) incubation with 4‐OHT, GFP^+^ cells indicate Cre‐recombinase activity. (e) Time course of GFP^+^ area extracted from images obtained with IncuCyte SX5 microscope taken every 2 h. (f) Quantification analysis of GFP^+^ area from cells treated with 4‐OHT for 120 h. Data are triplicates from independent experiments; paired Student's *t*‐test, *P < 0.0001. Scale bar, 100 μm.

**FIGURE 6:**
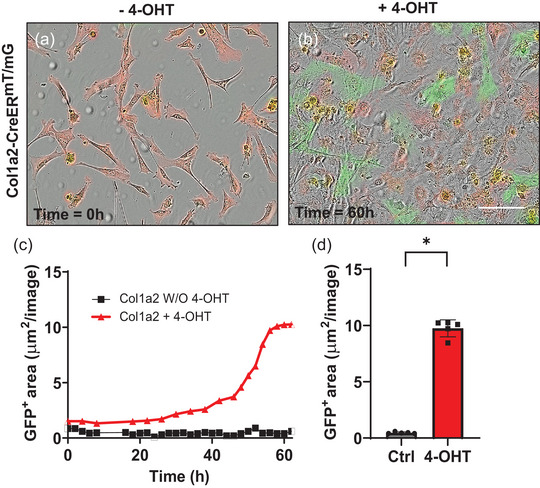
In vitro Col1a2‐CreER^mT/mG^ activation with 4‐OHT treatment in primary adult cardiac fibroblast. (a) Micrographs of Col1a2‐CreER^mT/mG^ extracted from live cardiac fibroblast treated with 4‐OHT. Phase and fluorescent image at ‘time 0’ before adding 4‐OHT. (b) Micrographs of Col1a2‐CreER^mT/mG^ taken 60 h after 4‐OHT treatment. (c) Time course of GFP^+^ from cells treated with 4‐OHT compared to untreated cells. (d) Data extracted from three independent experiments; Student's *t*‐test compared to control, *P < 0.0001. Scale bar, 100 μm.

Once metabolized, TAM is transformed into its active form, 4‐OHT, and direct i.p. injection of 4‐OHT instead of the TAM shows fewer side effects (Heinen et al., 2021). While this needs further confirmation, we tested the ability of 4‐OHT to induce gene recombination in vitro and assessed its potential toxicity. MEF cells were treated with a range of 4‐OHT concentrations for 72 h. Cell proliferation was measured by live‐cell imaging (IncuCyte) (Figure 5a,b). Proliferation was not affected by low concentrations of 4‐OHT up to 5 μM (*P* = 0.895). However, higher TAM concentrations halted cell proliferation and were toxic to the cells (Figure 5a,b, *P* = 0.0001). MEFs isolated from Col1A2‐CreER^mT/mG^ mice showed that 4‐OHT at 5 μM induces efficient genetic recombination indicated by the cells’ converting mTomato fluorescence into GFP^+^ fluorescence (Figure 5c–f, *P* = 0.0001). In contrast to the MEFs, cultures of adult cardiac fibroblasts isolated from the Col1a2‐CreER^mT/mG^ mice showed no recombinant cells at baseline (Figure 6a). However, in the presence of 4‐OHT, 50% of the cell population was GFP^+^ after 3 days of incubation with 4‐OHT (Figure 6b,c, *P* = 0.0001). These data show that 4‐OHT action depends on the cellular developmental stage and has a toxic effect on cell growth that can be avoided if used at low concentrations.

## 4 DISCUSSION

We tested the long‐term impact of a TAM citrate‐enriched diet and showed that laboratory mice did not fully adapt to the new diet. Signs of a starvation‐like phenotype were associated with changes in body composition. Occasional death occurred in the first 10 days of the diet. Mice that lived past the 10‐day mark had a high chance of handling a prolonged TAM diet but showed growth arrest and detrimental effects on cardiac function and heart morphology even without Cre‐recombinase expression. We found that mice fed with the TAM diet showed successful in vivo gene recombination but exhibited worse experimental outcomes when combined with other stressors like chronic MI. 4‐OHT can efficiently induce gene recombination in vitro but was associated with dose‐dependent cytotoxicity.

TAM has been extensively used for DNA editing in vitro and in vivo with the assumption that TAM and the oil used as vehicle are inert compounds. However, the number of reports describing the side effects of TAM in mice has increased considerably in recent years. Voluntary oral administration of TAM has emerged as safe and effective for inducing gene recombination in transgenic mice (Kiermayer et al., 2007; Yoshinobu et al., 2021). However, in our experimental set‐up, commercially available TAM‐enriched pellets caused significant weight loss and death. Mice that survived the 4 weeks of TAM treatment appeared fragile, with physical signs of starvation. TAM mimics oestradiol's effects on food intake (Wade & Heller, 1993), and it is well established that oestradiol reduces food intake and body weight, possibly via the activation of the oestrogen receptors (Asarian & Geary, 2006; Eckel, 2011). Therefore, the impact of TAM on mouse body weight and composition may be more complex than just food avoidance.

Our results showing transient adverse effects of TAM on cardiac function align with those from other reports of temporary adverse effects of TAM on the heart (Heinen et al., 2021; Rouhi et al., 2022). However, the causes of these negative effects were attributed to both the overexpression of the Cre and the high TAM dosage (Hall et al., 2011). Our work shows that TAM alone can decrease cardiac function and alter the organ anatomy even in the absence of Cre‐recombinase expression. Our results are supported by previous work showing that TAM and its derivatives decreased contraction amplitude, slowed relaxation and decreased Ca^2^⁺ transient amplitude when applied directly on isolated cardiomyocytes (Asp et al., 2013).

TAM is widely used for the treatment of breast cancer in women and men (Eggemann et al., 2018; Fisher et al., 1989). Several clinical trials examining the impact of chronic tamoxifen use on cardiac function concluded that TAM administration does not affect the rate of cardiovascular events in healthy women or women with pre‐existing coronary heart disease (Grainger & Schofield, 2005). However, a higher risk of cardiovascular disease‐related death associated with TAM use was reported (Greenlee et al., 2022). Our results should not be used to extrapolate any effect of TAM use in humans. In addition to potential inherent biological differences between humans and mice, in the latter, the serum concentration of TAM required to induce gene recombination can be 50–500 times higher than the concentration used for therapeutic use in humans (Donocoff et al., 2020)

Previous work using the TAM‐enriched diet has shown gene recombination in several tissues in physiological and pathological models. However, whether the TAM diet influenced the study outcome was not addressed. Our study shows that exposure to a TAM diet combined with MI successfully induced gene recombination in the myofibroblasts near and at the injury site. However, given the body weight loss associated with mouse frailty, it was unsurprising to observe high mortality in TAM‐fed mice post‐surgery. Therefore, a TAM diet can alter a study's outcome, primarily when used with other detrimental interventions that recapitulate human diseases.

Our study shows that MEFs can be exposed to relatively high concentrations of 4‐OHT but require 3 days before reaching maximal gene recombination. In contrast, adult cardiac fibroblasts showed maximal GFP expression much earlier, suggesting that the efficacy of 4‐OHT is development‐specific. We showed that 4‐OHT has dose‐dependent antiproliferative properties on fibroblasts. The antiproliferative effect of 4‐OHT in vitro has been reported (Buelow & Scharenberg, 2008); 4‐OHT exerts antiproliferative activity by increasing apoptosis due to reduced Bcl‐2 expression and decreased cell proliferation by altering the cell cycle kinetics (Cameron et al., 2000; Osborne et al., 1983). This is important, as TAM and 4‐OHT are often used in models where cell proliferation is a readout to assess the role of the targeted gene.

Of note, while the data represented here were obtained from a mouse strain often employed in studies where Cre‐induced recombination by TAM is required, our results cannot be extended to other mouse strains or across species because the genetic background may alter the response to the TAM diet and the resulting phenotype. We used naturally colored TAM‐enriched pellets in this study; however, pellets with different TAM concentrations, colors, and compositions are commercially available. TAM food pellets come with warnings and guidelines to minimize food avoidance. For instance, the TAM‐enriched pellet used in our experiments contained 5% more sucrose than the control diet, which may affect the body composition independently of TAM alone.

TAM400 offers a mid‐range TAM dosage and it is the most popular concentration reported in food pellets. We did not test other concentrations, but increasing or decreasing TAM concentration in the food pellets would be a trade‐off between gene recombination efficiency and toxicity in the animals. In our experimental set‐up, mice that survived the initial exposure reversed the adverse effects of the TAM diet after 4 weeks of a regular diet. We did not measure daily food intake. Therefore, we cannot discriminate between the impact of food avoidance and that of TAM. However, our results are supported by a significant amount of research that has described a similar impact of TAM or starvation on cardiac function (Koitabashi et al., 2009). Although our data rely on robust statistical analysis with power higher than 80%, a larger sample size may reduce the chances of a type II error.

Overall, TAM and 4‐OHT are invaluable tools to induce gene recombination in vivo and in vitro. However, while the voluntary oral administration of TAM avoids some side effects attributable to acute TAM and oil i.p. injections, it introduces variables that could hamper the interpretation of the results and the experimental outcome and potentially affect the function and role of the gene/protein of interest. Our findings emphasize the need for caution when using these models. When possible, a period of recovery from the diet should be allowed. When choosing the administration routes of TAM for gene recombination, its potential effects on the results' outcome should be carefully considered.

## AUTHOR CONTRIBUTIONS

Salvatore Mancarella conceived and designed the research; Janith Halpage and Patricia DaSilva Pantoja performed the experiments, analysed the data, and prepared the figures; Salvatore Mancarella provided experimental guidance and suggestions for revision, edited, and approved the final version of the manuscript. All authors have read and approved the final version of this manuscript and agree to be accountable for all aspects of the work in ensuring that questions related to the accuracy or integrity of any part of the work are appropriately investigated and resolved. All persons designated as authors qualify for authorship, and all those who qualify for authorship are listed.

## CONFLICT OF INTEREST

The authors declare no conflicts of interest.

## Data Availability

The data presented in this study are available on request.
